# Prognosis of lung cancer patients followed in the intensive care unit: A cross-sectional study

**DOI:** 10.5578/tt.20239917

**Published:** 2023-06-13

**Authors:** Ş.N. ÖZPINAR, A. G. KAYA, M. ÖZ, S. EROL, F. ARSLAN, A. ÇİLEDAĞ, A. KAYA

**Affiliations:** 1 Department of Radiology, Eskişehir Osmangazi University, Eskişehir, Türkiye

**Keywords:** Lung carcinoma, critical care, survival, mortality

## Abstract

**ABSTRACT:**

Prognosis of lung cancer patients followed in the intensive care unit: A cross-sectional study

**Introduction:**

Lung cancer is the most common solid organ malignancy requiring intensive care unit (ICU) admission. For many years, lung cancer patients were not considered in the priority patient category for admission to ICU because of their high mortality rate and poor response to therapy. Considering the developments in treatment modalities, we aimed to reevaluate the prognosis of patients with lung cancer in the ICU.

**Materials and Methods:**

Patients characteristics, date of diagnosis, the reason for ICU admission, the stage of cancer, histopathological type, history of chemotherapy, radiotherapy, or surgery for cancer, and APACHE-II and Charlson comorbidity index (CCI) were recorded retrospectively

**Results:**

A total of 100 patients had a mean age of 69.7 ± 9.0 years. Among these patients, 18% had small cell lung cancer, while 82% had non-small cell lung cancer. The in-hospital mortality rate was 69% for all patients, while among those discharged from the ICU, the first 6-month mortality rate was 58.1%. The median survival time was 8.2 months. Advanced age, the need for mechanical ventilation, the need for vasopressors, a high APACHE -II, and the CCI all reduced survival in multivariate analysis, whereas chemotherapy and surgical history improved survival.

**Conclusion:**

Patients admitted to the ICU with lung cancer continue to experience a high mortality rate. However, identifying the factors that are associated with survival can be crucial in establishing care plans and prioritizing ICU admission for further therapy.

## Abstract (tr)

**ÖZ:**

Yoğun bakım ünitesinde izlenen akciğer kanseri hastalarının prognozu: Kesitsel çalışma

**Giriş:**

Akciğer kanseri, yoğun bakım ünitesine (YBÜ) yatış gerektiren en yaygın solid organ malignitesidir. Uzun yıllar boyunca akciğer kanseri hastaları, yüksek ölüm oranları ve tedaviye zayıf yanıt vermeleri nedeniyle YBÜ'ye kabul için öncelikli hasta kategorisinde değerlendirilmemiştir. Tedavi modalitelerindeki gelişmeleri göz önünde bulundurarak yoğun bakım ünitesinde yatan akciğer kanserli hastaların prognozunu yeniden değerlendirmeyi amaçladık.

**Materyal ve Metod:**

Hastaların özellikleri, tanı tarihleri, yoğun bakıma yatış nedenleri, kanserin evresi, histopatolojik tipi, kanser nedeniyle kemoterapi, radyoterapi veya cerrahi öyküsü, APACHE-II ve Charlson komorbidite indeksi (CCI) retrospektif olarak kaydedildi.

**Bulgular:**

Toplam 100 hastanın yaş ortalaması 69,7 ± 9,0 idi. Hastaların %18’inde küçük hücreli akciğer kanseri bulunurken, %82’sinde küçük hücreli dışı akciğer kanseri vardı. Hastane içi ölüm oranı hastaların %69’u iken, yoğun bakım ünitesinden taburcu olanlarda ilk altı aylık ölüm oranı %58,1 oldu. Ortalama hayatta kalma süresi 8,2 aydı. Çok değişkenli analizde ileri yaş, mekanik ventilasyon ihtiyacı, vazopresör ihtiyacı, yüksek APACHE-II ve CCI’nin tümü sağkalımı azaltırken, kemoterapi ve cerrahi öyküsü sağkalımı arttırdı.

**Sonuç:**

Akciğer kanseri nedeniyle yoğun bakıma kabul edilen hastalarda ölüm oranı hala yüksektir. Bununla birlikte, hayatta kalma ile ilişkili faktörlerin belirlenmesi, bakım planlarının oluşturulmasını ve ileri tedavi için yoğun bakım ünitesine yatışa öncelik verilmesini sağlayabilir.

## Introduction

In 2018, lung cancer was diagnosed in approximately 2.1 million patients worldwide and caused the death of an estimated 1.8 million patients ( 1 ). The presence of weight loss, disease stage, and performance status have all been associated with survival in patients with lung cancer ( 2 ). Although survival has improved over many years in cancer patients requiring intensive care ( 3 , 4 , ), lung cancer has the highest ICU mortality rate among solid tumors ( 5 , 6 ). The indication for admission to the ICU, rather than the prognosis of the underlying malignancy, determines the prognosis of patients followed up in the ICU ( 7 ). Previous studies have shown that acute respiratory failure ( 8 , 9 ), sepsis ( 8 , 10 ), organ dysfunction involving more than two organs ( 9 ), the need for mechanical ventilation (8,11), the need for vasopressors ( 12 ), ECOG performance status ( 13 ), and the presence of metastatic ( 11 ) or progressive disease ( 9 ) are the main predictors of poor prognosis in this population.

In this study, the in-hospital and sixth-month mortality status of lung cancer patients followed in the ICU were assessed to determine the prognosis of these patients.

## MATERIALS and METHODS

### Patient Selection and Inclusion Criteria

The study included patients who were over 18 years old and had histopathologically confirmed lung cancer, and who had stayed in the intensive care unit (ICU) for a minimum of 24 hours. Patients under 18 years of age, those hospitalized in the ICU for less than 24 hours, and those who had not had a recurrence in at least five years of follow-up after completing lung cancer therapy were excluded from the study.

### Data Collection

The following information was recorded for the participants: age, gender, age at lung cancer diagnosis, duration of ICU hospitalization, cancer stage, histopathological type of lung cancer, history of chemotherapy, radiotherapy, or cancer-directed surgery since the diagnosis of lung cancer, reasons for ICU admission, APACHE-II score, Charlson Comorbidity Index (CCI), need for renal replacement therapy, need for vasopressors, and mortality during hospitalization or mortality rates in the first three and six months after hospitalization.

The mask images of the labeled regions were generated within the computer science department of our faculty and saved using the same names. Subsequently, the dataset was divided into three distinct groups, namely training, validation, and testing, with proportions of 80%, 10%, and 10% respectively. The mixed-size images were resized to 512 x 512. By applying 50% zoom to the images, the regions to be segmented were enlarged as much as possible to fit the image. The clarity of the regions to be segmented was increased by applying contrastlimited adaptive histogram equalization.

In this study, lung cancer was classified histopathologically into non-small cell lung cancer (NSCLC) and small cell lung cancer (SCLC). Advanced disease was defined as the presence of Stage IV disease in patients with a diagnosis of NSCLC and the presence of extensive disease in patients with a diagnosis of SCLC. Since the diagnosis of lung cancer, the patient’s history of chemotherapy (CT), radiation (RT), and previous surgery for lung cancer were recorded.

The reason for admission of patients to ICU was classified into sepsis, respiratory reasons, cardiac reasons, neurological reasons, post-cardiopulmonary arrest, and other reasons. Whether mechanical ventilation (MV) was used or not, and if it was, whether the MV was invasive or non-invasive, was documented during the intensive care follow-up.

### Statistical Method

For quantitative variables, descriptive statistics were presented as mean, standard deviation (for parametric continuous variables), or median and minimummaximum (for nonparametric continuous variables), and for qualitative variables, frequency (percentage). The Independent Samples t-test or Mann-Whitney U test for quantitative variables and Chi-square or Fisher exact test for qualitative factors were used to compare clinical and demographic data from surviving and non-surviving lung cancer patients. The Kaplan-Meier method was used to calculate survival rates and create survival curves. The cox proportional hazard model was used to assess risk factors for mortality and survival time, and logistic regression Analysis was used to determine independent prognostic variables present during ICU admission. The statistical significance level was assumed to be 0.05, and the analysis was carried out with the help of the Statistical Package for Social Sciences (SPSS, Version 15.0, Chicago, IL.).

## RESULTS

The mean age of 100 patients with lung cancer followed up in the ICU was 69.7 ± 9.0 years (min 46-max 92), and 79% of the patients (n= 79) were male. When the patients were categorized based on their histological forms of lung cancer, 18% (n= 18) had SCLC, and 82% (n= 82) had NSCLC. All SCLC patients had extensive disease. According to TNM staging, one of 82 patients with NSCLC was at stage I, four were at stage III, and 77 were at stage IV. Advanced disease was defined as having extensive disease in the SCLC group and stage IV disease for NSCLC. According to this categorization, 95% of patients (n= 95) were classified as having advanced disease. Following a lung cancer diagnosis, 57% (n= 57) of the 100 patients in the study received chemotherapy, 49% (n= 49) received radiotherapy, and 17% (n= 17) had a history of lung cancer surgery. 57% (n= 57) of a total of 100 patients were referred to the ICU for respiratory pathologies, 16% (n= 16) after cardiopulmonary arrest during follow-up in the emergency department or other in-hospital services, 11% (n= 11) for sepsis, 11% (n= 11) for neurological pathologies and 5% (n= 5) for cardiovascular pathologies. Eighty-four percent (n= 84) of the patients required MV support during the ICU followup. In patients who required MV, non-invasive (NIMV) treatment was administered to 29.8% (n= 25), and invasive (IMV) treatment to 70.2% (n= 59). 47% (n= 47) of the patients received vasopressors and 17% (n= 17) received renal replacement therapy during their ICU stay. The median APACHE-II score was 24.5 (min 5-max 49) when evaluating the patients’ illness severity during their time in intensive care. Additionally, the CCI was calculated as 7.8 ± 1.8, falling within the range of 6 to 15. While 69% (n= 69) of patients died during their ICU stay, 31% (n= 31) were discharged. When the patients’ mortality status was assessed after discharge from the ICU, 25.8% (n= 8) died within the first three months, and 32.3% (n= 10) died between three and six months. All patients who died in the ICU were due to lung cancer and cancer-related complications.

When the demographic and clinical characteristics of patients who died and those who survived in the ICU were examined, the prevalence of advanced disease (87.1% vs 98.6%, p= 0.031) and the requirement for MV were lower in the survivors (51.6% vs 98.6%, p< 0.001). When patients underwent MV, those who required IMV had a lower survival rate (6.7% vs 48%, p< 0.001) than those who required NIMV. Vasopressor requirement (6.5% vs 65.2%, p< 0.001) was also lower in survivors. All 45 patients who required renal replacement therapy died in ICU. Furthermore, surviving patients had a lower APACHEII score [12 (5-33) vs 26 (9-49), p< 0.001]. When evaluating patients with ICU mortality and survivors regarding ICU admission indications, respiratory pathologies were seen more frequently in patients with mortality (59.6% vs 40.4%, p= 0.020)

The median survival of all patients included in the study was 8.2 months (std error= 1.6, 95% CI 4.9- 11.4 months) (Figure 1A). The survival of patients discharged from the ICU was also assessed independently. The median survival time was 9.2 months (std error= 1.7, 95% CI 5.8-12.5 months) ( Figure 1B ).

**Figure 1A:**
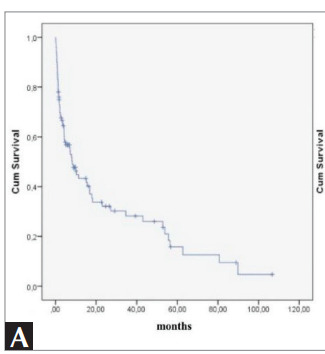
Graph of the ROC analysis (ROC, receiver operating characteristic. AUC: Area under the curve

**Figure 1B:**
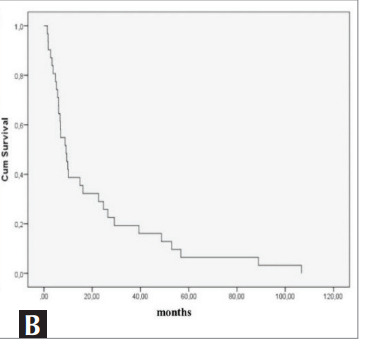
Computed tomographic pulmonary angiography examination revealed filling defects consistent with acute pulmonary embolism in the pulmonary arteries and segment branches of the lower lobes of both lungs in a 63 year-old male patient. Segmentation of the areas consistent with the detected acute pulmonary embolism was performed.

Patients with a history of chemotherapy (p<0.001) after diagnosis, a history of radiotherapy (p= 0.015), a history of lung cancer surgery (p= 0.034); patients who do not require MV (p< 0.001) during hospitalization in the intensive care unit, use of NIMV treatment in patients who needed MV (p= 0.017), and no need for vasopressors (p= 0.001) were found to be associated with survival times when the Kaplan-Meier test was used to analyze the factors related to survival times. Gender (p= 0.096), a disease with ICU indication (p= 0.327), and the need for renal replacement therapy (p= 0.199) were shown not to affect survival ( Table 2 ).

Age (HR= 0.92, 95% CI 0.88-0.96, p<0.001), the requirement for MV (HR= 0.04, 95% CI 0.01-0.51, p= 0.013), the need for renal replacement therapy (HR= 0.042, 95% CI 0.20-0.86, p= 0.018), a higher APACHE-II score (HR= 0.95, 95% CI 0.92-0.98, p= 0.001), and a higher CCI score (HR= 0.75, 95% CI 0.47-0.98, p= 0.026) all reduced survival in-hospital. Besides, in-hospital survival was increased by a history of chemotherapy (HR= 6.01, 95% CI= 2.97- 12.48, p< 0.001) and previous lung cancer surgery (HR= 2.35, 95% CI 1.10-5, 03, p= 0.027) (Table 3). For the out-of-hospital survival, the history of the surgery was found to increase the survival (HR= 5.23, 95% CI 1.09-24.94, p= 0.038). Also, a one-unit increase in the Charlson comorbidity score reduced mortality (HR= 0.53, 95% CI 0.34-0.83, p= 0.005). Out-of-hospital survival was not affected by age (p= 0.985), gender (p= 0.100), presence of advanced disease (p= 0.266), chemotherapy history (p= 0.660), radiotherapy history (p= 0.725), APACHE II score (p= 0.555), or MV need (p= 0.554) ( Table 4 ).

## DISCUSSION

Most cancer patients encounter life-threatening complications due to treatment, comorbidities, or tumor effects. In previous years, the idea of not prioritizing the admission of patients with advanced cancer to the ICU was common ( 14 ). Several studies have found that survival rates in this population have increased in recent years ( 15 , 16 ). Oncology and intensive care developments are thought to have contributed to improving survival rates ( 17 ). Our study showed a history of chemotherapy and surgery due to lung cancer, the need for MV and renal replacement therapy, and APACHE II and CCI scores were associated with survival in ICU for patients with lung cancer.

The present study showed that 69% of lung cancer patients admitted to the ICU died during their stay, and 87% died within the first six months. Different mortality rates were given in previous studies, including patients with lung cancer followed in the ICU ( Table 5 ). Multi-center research by Slatore et al. analyzed almost 50000 lung cancer patients and reported in-hospital mortality of 25%. The authors indicated their study patients with milder diseases admitted to intermediate intensive care units, that is, who did not require life support service, were cited as a critical factor in the reduced in-hospital mortality rate ( 12 ). ICU mortality was found to be 22% in another research conducted by Adam and Soubani,

**Table 1:** Sociodemographic and clinical characteristics of the patients

Characteristics	
Age (year) mean ± SD	69.7 ± 9.0
Gender (man) n (%)	79 (79)
Histological type n (%)	
Small cell	18 (18)
Non-small cell	82 (82)
Advanced disease* n (%)	95 (95)
Chemotherapy history n (%)	57 (57)
Radiotherapy history n (%)	49 (49)
Operation history n (%)	17 (17)
ICU indication n (%)	
Respiratory pathologies	57 (57)
Post cardiopulmonary arrest	16 (16)
Sepsis	11 (11)
Neurological pathologies	11 (11)
Cardiovascular pathologies	5 (5)
Need for MV n (%)	84 (84)
MV type n (%)	
NIMV	25 (29.8)
IMV	59 (70.2)
Need for vasopressors n (%)	47 (47)
Need for RRT n (%)	17 (17)
APACHE-II median (min-max)	24.5 (5-49)
CCI mean ± SD	7.8 ± 1.8

*Patients with extensive disease for SCLC, patients with stage IV for NSCLC. ICU: Intensive care unit, APACHE: Acute physiology and chronic health evaluation,CCI: Charlson comorbidity index, MV: Mechanical ventilation, RRT: Renal replacement therapy, NIMV: Noninvasive mechanical ventilation, IMV: Invasive mechanical ventilation.

and it was stated that 49% of the patients required mechanical ventilation, and the study’s selection of patients who might benefit from the ICU could have resulted in a bias.

Furthermore, the authors suggested that the difference between studies could be due to the inclusion of younger patients and the low rate of patients with distant metastases ( 15 ). Puxty et al. observed that in-hospital mortality was 58% and six-month mortality was 68.8% in a multicenter study of newly diagnosed lung cancer patients who were followed up in the ICU. The lower mortality rate in this study compared to our study might be related to the fact that the patients included in the study were newly diagnosed lung cancer cases, and the early-stage lung cancer rates were higher than in our study ( 15 ). Ninety-five percent of our study group had advanced disease (stage IV for NSLC, extensive disease for SCLC), and the high rate of progressive disease may be the reason for our overall higher mortality rate.

Our study revealed that the need for mechanical ventilation support during ICU follow-up is associated with decreased survival. Similarly, several studies have shown that lung cancer patients admitted to the ICU who require MV have a poorer prognosis than those who do not ( 8 , 9 , 10 , 11 ). Mechanical ventilation, the use of vasopressors, organ failure, and the presence of a septic condition all lowered survival in 105 patients with lung cancer admitted to the ICU, according to a study by Anisoglou et al. ( 10 ). Andrejak et al. conducted a study involving 76 patients with lung cancer, where they observed that the requirement for mechanical ventilation (MV), the need for vasopressors, and the presence of thrombocytopenia were all associated with reduced survival ( 18 ). According to Adam and Soubanis’ study ( 15 ), mechanical ventilation, a high APACHE-II score, the use of vasopressors, growth in blood culture, elevated serum lactate levels, and the presence of two or more organ dysfunctions were all associated with lower mortality. According to a national study by Lai et al. of 17.687 lung cancer patients admitted to the ICU, advanced age and organ failure contributed to 1-year mortality. Still, mortality was reduced in those undergoing chemotherapy and recent lung cancerrelated surgery ( 19 ).

The 2018 study by Louie et al. found that ICU discharge incidence was lower in patients who received radiotherapy, and mortality was higher in patients who received radiotherapy. However, the study reported that radiotherapy was used in a minimal number of lung cancer patients, and these patients who received radiotherapy required more mechanical ventilation support. Furthermore, it has been observed that the utilization of radiotherapy and the number of treatment sessions can vary significantly across different medical centers. After adjusting for these factors, survival was no different in patients who received radiotherapy than in those who did not ( 20 ). Moreover, there are data that radiation therapy can cause radiation pneumonia and acute respiratory failure, which may increase mortality ( 18 ). The association between the history of

**Table 2:** Survival time analysis for all patients admitted to the intensive care unit

	Median survival time (months)	Standard error	95% confidence interval	p
Overall survival time	8.2	1.6	4.9-11.4	
Gender				
Female	8.0	3.5	1.0-14.9	0.832
Male	8.7	2.1	4.5-12.8	
Histological type				
Small cell	3.5	2.8	0-9.1	0.09
Non-small cell	8.7	3.9	1.0-16.4	
Chemotherapy history Yes	16.8	3.9	9.0-24.6	<0.001
Radiotherapy history Yes	10.2	4.0	2.2-18.2	0.065
Operation history Yes	34.5	13.0	9.0-60.0	0.034
ICU indication				
Respiratory pathologies	10.2	5.5	0-21.0	0.327
Post cardiopulmonary arrest	4.2	0.1	3.9-4.4	
Sepsis	8.7	6.1	0-20.7	
Neurological pathologies	11.4	6.5	0-24.2	
Cardiovascular pathologies	2.4	0.6	1.1-3.6	
Need for MV Yes	4.9	1.7	1.4-8.3	<0.001
MV type				
Noninvasive IMV	15.1	5.1	5.0-25.2	0.017
Invasive MV	4.1	0.9	2.2-5.9	
Need for vasopressors Yes	4.1	1.1	1.8-6.5	0.001
Renal replacement therapy Yes	4.3	0.7	2.9-5.6	0.199

*Kaplan Meier survival analysis, the analysis used Long-rank, Breslow, or Tarone-Ware tests. ICU: Intensive care unit. MV: Mechanical ventilation.

radiotherapy and survival could not be demonstrated in our study.

In our study, respiratory pathologies were the most common reason for ICU admission. Similar to our findings, respiratory pathologies have previously been described as the most common reason for lung cancer admission to the ICU in various studies ( 21 , 22 ).

In our study, patients with a history of chemotherapy demonstrated a higher survival rate. However, it is important to note that the specific type and duration of chemotherapy were not investigated in our study.

Furthermore, in our analysis, we considered the patients’ history of chemotherapy prior to their admission to the intensive care unit (ICU). A study by Nasser et al. revealed that cisplatin and pemetrexed treatment in the ICU in NSCLC patients produced positive results (23). According to Chen et al. study, even chemotherapy given during a patient’s admission to the ICU improved short-term survival in lung cancer patients who had not previously received treatment ( 24 ).

Our findings indicated that the presence of factors such as the need for mechanical ventilation (MV), the

**Table 3:** Multivariant Cox regression analysis of factors affecting the survival in intensive care unit

	B	HR	95% CI	p
Age	-0.078	0.92	0.88-0.96	<0.001
Gender, (man)	-0.203	0.81	0.40-1.63	0.565
Histological type, small cell	0.468	0.62	0.32-1.19	0.154
Advanced disease (+)	0.760	2.13	0.17-26.55	0.554
Chemotherapy history (+)	1.808	6.01	2.97-12.48	<0.001
Radiotherapy history (+)	-0.139	0.87	0.45-1.67	0.676
Operation history (+)	0.856	2.35	1.10-5.03	0.027
MV need (+)	-3.172	0.04	0.01-0.51	0.013
Vasopressor need (+)	-0.432	0.64	0.35-1.17	0.154
RRT need (+)	-0.855	0.42	0.20-0.86	0.018
APACHE II score	-0.051	0.95	0.92-0.98	0.001
CCI	-0.229	0.75	0.47-0.98	0.026

MV: Mechanical ventilation, APACHE; Acute physiology and chronic health evaluation, CCI; Charlson comorbidity index, RRT: Renal replacement therapy. *Cox regression analysis, HR: Hazard ratio, CI: Confidence interval.

**Table 4:** Multivariant Cox regression analysis of factors affecting the survival out-of-hospital

	B	HR	95% confidence interval	p
Age	0.004	1.00	0.94-1.06	0.905
Gender, man	-0.013	0.98	0.26-3.62	0.985
Histological type, small cell	-1.250	0.28	0.06-1.27	0.100
Advanced disease (+)	-0.929	0.39	0.07-2.02	0.266
Chemotherapy history (+)	0.265	1.30	0.40-4.25	0.660
Radiotherapy history (+)	0.193	1.21	0.41-3.55	0.725
Operation history (+)	1.656	5.23	1.09-24.94	0.038
APACHE II score	-0.022	0.97	0.90-1.05	0.555
CCI	-0.622	0.53	0.34-0.83	0.005
MV need (+)	-0.283	0.75	0.29-1.92	0.554

Advanced disease: Patients with extensive disease for SCLC, stage IV for NSCLC APACHE: Acute physiology and chronic health evaluation, CCI: Charlson comorbidity index, MV: Mechanical ventilation. *Cox regression analysis, HR: Hazard ratio.

requirement for vasopressor treatment, the indication for ICU admission, and APACHE-II scores did not have a significant impact on the out-of-hospital survival of the patients. In contrast, the patients’ CCI and surgery history did. As a result, a patient’s history of cancer-related surgeries positively impacts survival both in and out of the ICU. On the contrary, comorbid conditions assessed with CCI may have a detrimental effect on patients’ survival both in and out of the ICU. When postoperative complications are effectively managed and surgeries are conducted in experienced medical centers, acceptable postoperative mortality rates have been reported in lung cancer patients, which significantly contribute to the patients’ prognosis ( 25 , 26 ). In prioritizing intensive care unit (ICU) admission, it is important to consider patients who have the potential to benefit from specific cancer-directed treatments. The higher survival observed in patients with a history of chemotherapy and surgery may be linked to their improved tolerance of complications related to treatment. Furthermore, it can be inferred that patients who receive specific

**Table 5:** Previous published data on lung cancer patients admitted to the ICU

	Number of patients	NSCLC (%)	Stage IV or extensive disease (%)	MV need (%)	Hospital mortality (%)
Ewer et al. (27)	46	65	NR	100	91
Boussat et al. (28)	57	92	47	91	75
Lin et al. (29)	81	77	59	100	85
Reichner et al. (11)	47	83	53	49	60
Soares et al. (9)	143	83	NR	70	59
Adam et al. (15)	139	69	40	49	40
Roques et al. (30)	105	83	NR	41	54
Bonomi et al. (8)	1134	100	55	14	33
Andrejak et al. (18)	76	65	59	75	65
Kim et al. (13)	95	84	76	90	78
Chang et al. (31)	143	88	25	93	74
The present study	100	82	95	84	69

MV: Mechanical ventilation, NSCLC: Non-small cell lung cancer, NR: Not reported.

cancer treatments tend to have a better overall condition. This is evident by their better performance and outcomes compared to those who do not receive such treatments.

Our study had some limitations. Our main limitation was that our study was retrospective. Because our patients had a significant level of comorbidity and were of advanced age, it was assumed that age and comorbidity could have influenced our research findings as confounding factors. Another limitation is that the study patients were not classified according to the combination of chemotherapy, radiotherapy, and surgery, which may also affect the prognosis.

As a result, the idea of not giving priority to the ICU for patients with lung cancer has lost its validity over time. With advances in inpatient treatment and ICU services, the ICU’s performance in this patient group can be further enhanced by developing ICU admission strategies, selecting patients who will benefit from ICU, and developing individualized ICU treatment approaches. A history of chemotherapy and surgery due to lung cancer, the need for MV and renal replacement therapy, and APACHE II and CCI scores were associated with the prognosis of ICU for patients with lung cancer.

## Ethical Committee Approval

This study was approved by Ankara University Human Researches Ethics Committee (Decision no: İ10-654-20, Decision date: 04.12.2020).

## Conflict of INTEREST

The authors declare that they have no conflict of interest.

## AUTHORSHIP CONTRIBUTIONS

Concept/Design: All of authors

Analysis/Interpretation: ŞÖ, AGK

Data acqusition: : All of authors

Writing: ŞÖ, AGK, AK

Clinical Revision: All of authors

Final Approval: All of authors
